# Low-Complexity Time-Domain Ranging Algorithm with FMCW Sensors

**DOI:** 10.3390/s19143176

**Published:** 2019-07-19

**Authors:** Xi Pan, Chengyong Xiang, Shouliang Liu, Shuo Yan

**Affiliations:** 1 School of Mechatronical Engineering, Beijing Institute of Technology, Beijing 100081, China; 2 Beijing Research Institute of Telemetry, Beijing 100076, China

**Keywords:** time-domain ranging, frequency-modulated continuous wave, low-complexity, beat frequency

## Abstract

A time-domain ranging algorithm is proposed for a frequency-modulated continuous wave (FMCW) short-range radar sensor with high accuracy and low complexity. The proposed algorithm estimates the distance by calculating the ratio of the beat frequency signal to its derivative and thereby eliminates the restriction of frequency bandwidth on ranging accuracy. Meanwhile, we provide error analysis of the proposed algorithm under different distances, integral lengths, relative velocities, and signal-to-noise ratios (SNRs). Finally, we fabricate FMCW sensor prototype and construct a measurement system. Testing results demonstrate that the proposed time-domain algorithm could achieve range error within 0.8 m. Compared with the conventional fast Fourier transform (FFT) estimation scheme, the proposed method performs ranging without the requirement of complex multiplications, which makes it reasonable to be implemented in real-time and low-cost systems.

## 1. Introduction

Frequency-modulated continuous wave (FMCW) radars have been widely used for short range measurements benefiting from their high ranging resolution. The FMCW proximity radar sensor was extensively applied in industrial community, such as liquid level measurements [1], direction of arrival (DOA) estimation [2], radar altimeters [3,4], mechanical vibrations [5], and biomedical measurements [6]. The FWCW sensor employs beat frequency signal to measure the distance [7] by mixing the transmitted signal with the received time-delayed signal. The discrete spectral components of the beat frequency are directly related to the signal propagation time, or equivalently, the distance between sensor and target. Therefore, most approaches in the literature exploit the frequency-domain signals to estimate a spectral component [8,9,10,11].

Distinct methods for estimating the spectral peak of beat frequency signal in frequency-domain have been proposed to improve the range resolution. The rough values of beat frequency were first estimated in [12] using discrete Fourier transform (DFT) for FMCW systems, where gradient search methods (GSMs) were employed in subsequent iterations to obtain the precise peak value. An additional local maximum peak near the global maximum was used by a curve-fitting device in [13] to obtain the range adjustment and improve the range accuracy for FMCW radars. A difference method using the eigenvalue-based multiple signal classification (MUSIC) algorithm was proposed in [14,15] to provide sharp spectral lines and high resolution spectral estimation. The spectral peak search methods mentioned above rely on the fast Fourier transform (FFT) or the MUSIC algorithm and usually suffer from high computational cost. The Chirp-Z transform (CZT) can refine the spectrum based on an interpolation technique with low computational complexity [16]. An optimized CZT algorithm was proposed in [17] to achieve increasing frequency resolution with an additional phase evaluation. Kim et al. [18] also proposed a low-complexity algorithm using two random beat signals to perform one-dimensional FFT for range detection.

Unlike those conditions presented in the above works, the ranging system and the target in our application are moving with a relative velocity and the range calculation time allowed is limited, requiring a real-time high-resolution estimation method. We propose a novel ranging algorithm exploiting the time-domain signal instead of the conventional estimation method in the frequency-domain. The main contributions of this paper are as follows:

- We proposed a time-domain ranging algorithm, essentially by calculating the ratio of the beat frequency signal to its derivative, and analyzed the inherent error of the proposed estimation in the case of spectral dispersion. Results show that the ranging resolution is unrestricted by the frequency bandwidth of the modulating signal.
- After fabricating the FMCW sensor prototype, we conducted experiments to validate the proposed ranging scheme with ranges r = 4–18 m. Measured range errors exhibit high ranging resolution below 0.8 m and periodical characteristics for the increasing range.
- We also provided complexity analysis which indicated that the time-domain ranging algorithm has lower computational complexity without the requirement of complex multiplications, compared with conventional frequency-domain methods.

The rest of this paper is organized as follows: In Section 2, we introduce the ranging model with beat frequency signal and then propose a time-domain ranging method with detailed theoretical derivations. In Section 3, we investigate the ranging errors under different SNRs, moving speeds, integral numbers, and provide the computation complexity analysis. The extensive experiments will be conducted in Section 4 to validate our novel ranging method. Finally, conclusions are drawn in Section 5.

## 2. Time-Domain Ranging Algorithm with Beat Frequency

In this section, we commence to introduce the time-domain expression of the beat frequency signal. Then, the time-domain ranging algorithm will be derived with some direct approximations.

### 2.1. Principle of Beat Frequency Signal

**Figure 1:**
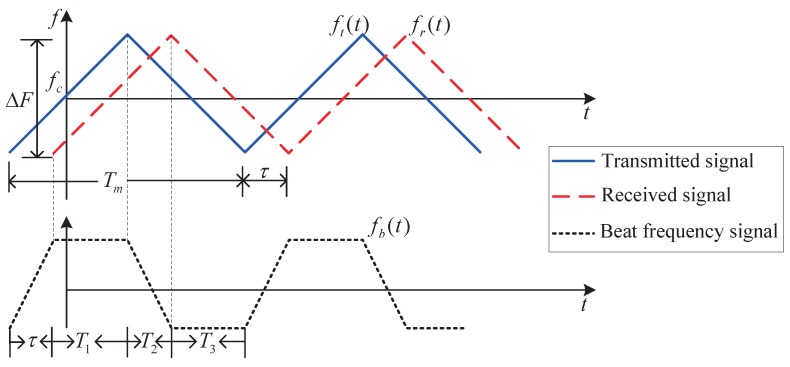
Instantaneous frequency of the transmitted and received signals for relatively stationary sensor and target.

The periodic modulation of the beat frequency signal is shown in Figure 1, where $f_{c}$ is the carrier frequency, ΔF is the frequency bandwidth of the modulating signal, $T_{m}$ is the modulation period, $T_{1}$ and $T_{3}$ are the regular intervals, $T_{2}$ is the odd interval, and τ is the propagation delay of the received signal. The transmission frequency deviates from $f_{c}-\Delta F/2$ to $f_{c}+\Delta F/2$.

**Figure 2:**
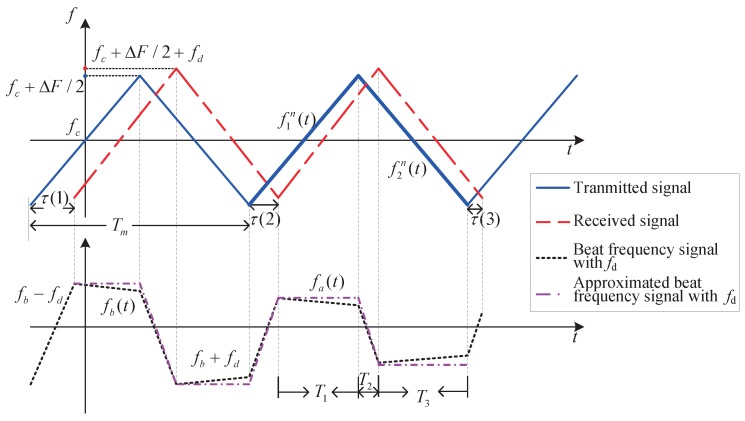
Instantaneous frequency of the transmitted and received signals with relative motion between sensor and target.

The transmitted signal $f_{t}\left(t\right)$ is modulated by a triangular waveform. When the sensor and the target are relatively stationary, the instantaneous frequency of the beat frequency signal could be described as $f_{b}\left(t\right)$ in Figure 1. If there is relative motion, the beat signal will be slightly different, taking account of the Doppler shift effect $f_{d}$, as given in Figure 2, where the target and the sensor are moving towards each other and the frequency of the received signal will become $f_{r}\left(t\right)+f_{d}$. The relative motion between target and sensor would lead to a slight change of the range during the regular interval, which finally brings a reduction on the regular interval frequencies of the beat frequency signal. Compared with the absolute value of the beat frequency, this ranging variation is negligible. Therefore, each regular interval frequency can be approximated as its initial frequency. The beat signal can be approximated by $f_{a}\left(t\right)$, as indicated in Figure 2.

**Figure 3:**
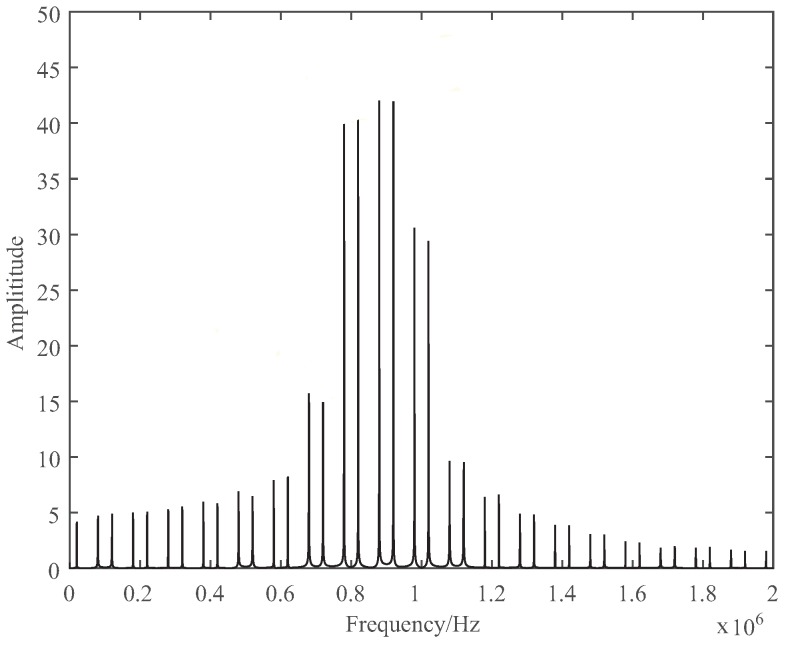
Discrete-spectrum of beat frequency signal with $F_{m}$ = 100 kHz.

The beat spectrum is discrete and the spectral grid is $F_{m}$ ($F_{m}=\frac{1}{T_{m}}$), as shown in Figure 3, where $F_{m}$ = 100 kHz. The theoretical range resolution is given by $\Delta R=\frac{c}{4\Delta F}$ [7] for FFT estimation with some type of window, where *c* is the speed of light. This range resolution is directly proportional to ΔF, and it could be improved only by increasing the frequency bandwidth of the modulating signal, which is restricted by hardware resources and computation cost of implementation.

### 2.2. Time-Domain Expressions of Beat Frequency

The instantaneous frequencies $f_{1}^{n}\left(t\right)$ and $f_{2}^{n}\left(t\right)$ of the rising and falling intervals of the transmitted signal can be expressed as

(1) $$\left\{\begin{array}{c} f_{1}^{n}\left(t\right)=f_{c}+\beta \left(t-nT_{m}\right),\;\;\;\;\;\;\left(n-1/4\right)T_{m}\leq t<\left(n+1/4\right)T_{m})\\ f_{2}^{n}\left(t\right)=f_{c}+▵F-\beta \left(t-nT_{m}\right),\;\left(n+1/4\right)T_{m}\leq t<\left(n+3/4\right)T_{m}), \end{array}\right.$$

where $\beta =\frac{2\Delta F}{T_{m}}$. The instantaneous phases of the transmitted signal at two intervals following $\varphi =2\pi \int_{0}^{t}f\left(t\right)dt$ are derived as

(2) $$\left\{\begin{array}{c} \varphi_{1}^{n}\left(t\right)=\frac{\beta t^{2}}{2}+\left(f_{c}-n\beta T_{m}\right)t+\frac{\beta n^{2}T_{m}^{2}}{2},\;\;\;\;\;\;\;\;\;\;\;\;\;\;\;\;\;\left(n-1/4\right)T_{m}\leq t<\left(n+1/4\right)T_{m})\\ \varphi_{2}^{n}\left(t\right)=-\frac{\beta t^{2}}{2}+\left(f_{c}+▵F+n\beta T_{m}\right)t-\frac{\beta {\left(n+1/2\right)}^{2}T_{m}^{2}}{2},\;\;\left(n+1/4\right)T_{m}\leq t<\left(n+3/4\right)T_{m}). \end{array}\right.$$

Therefore, the transmitted signal $s_{t}\left(t\right)$ can be written as

(3) $$s_{t}\left(t\right)\;=\;\left\{\begin{array}{c} u_{t}\cos\left\{2\pi \left[\frac{\beta t^{2}}{2}+\left(f_{c}-n\beta T_{m}\right)t+\frac{\beta n^{2}T_{m}^{2}}{2}\right]\right\},\;\;\;\left(n-1/4\right)T_{m}\leq t<\left(n\;+\;1/4\right)T_{m}\\ u_{t}\cos\left\{2\pi \left[-\frac{\beta t^{2}}{2}+\left(f_{c}+▵F+n\beta T_{m}\right)t-\frac{\beta {\left(n+1/2\right)}^{2}T_{m}^{2}}{2}\right]\right\},\left(n\;+\;1/4\right)T_{m}\leq t<\left(n\;+\;3/4\right)T_{m}, \end{array}\right.$$

where $u_{t}$ is the strength of the transmitted signal.

The difference between the transmitted and the received signals includes the propagation delay τ(t), the Doppler shift $f_{d}$, and the extra phase shift $\varphi_{r}$ induced by the reflective characteristics of the target. Since $v_{r}\ll c$, the time delay $\tau \left(t\right)=\frac{2R_{0}}{c}-\frac{2v_{r}t}{c}\approx \frac{2R_{0}}{c}=\tau$, where $R_{0}$ is the initial range within a regular interval and $v_{r}$ is the relative speed as the target approaching the sensor. Therefore, we can get the reflected signal $s_{r}\left(t\right)$ as

(4) $$s_{r}\left(t\right)\;=\;\left\{\begin{array}{c} u_{r}\cos\left\{2\pi \left[\frac{\beta {\left(t-\tau \right)}^{2}}{2}+\left(f_{c}+f_{d}-n\beta T_{m}\right)\left(t-\tau \right)+\frac{\beta n^{2}T_{m}^{2}}{2}\right]\right\},\;\;\;\left(n-1/4\right)T_{m}\leq t<\left(n\;+\;1/4\right)T_{m}\\ u_{r}\cos\left\{2\pi \left[-\frac{\beta {\left(t-\tau \right)}^{2}}{2}+\left(f_{c}+f_{d}+▵F+n\beta T_{m}\right)\left(t-\tau \right)-\frac{\beta {\left(n+1/2\right)}^{2}T_{m}^{2}}{2}\right]\right\},\left(n\;+\;1/4\right)T_{m}\leq t<\left(n\;+\;3/4\right)T_{m}. \end{array}\right.$$

The propagation delay τ is usually negligible with hundreds of nanoseconds, compared to the modulation period in the order of tens of microseconds. In these cases, the beat frequency can be expressed as

(5) $$s_{b}\left(t\right)\;=\;\left\{\begin{array}{c} u_{b}\cos\left\{2\pi \left[f_{c}\;+f_{d}+\;\left(\frac{2\Delta F}{T_{m}}-\frac{f_{d}}{\tau }\right)\left(t\;-\;nT_{m}\right)\;-\;\frac{\Delta F}{T_{m}}\tau -\frac{f_{d}}{\tau }nT_{m}\right]\tau \right\},\left(n-1/4\right)T_{m}\leq t<\left(n\;+\;1/4\right)T_{m}\\ u_{b}\cos\left\{2\pi \left[f_{c}\;+\;\Delta F+f_{d}\;-\;\left(\frac{2\Delta F}{T_{m}}+\frac{f_{d}}{\tau }\right)\left(t\;-\;nT_{m}\right)\;+\;\frac{\Delta F}{T_{m}}\tau -\frac{f_{d}}{\tau }nT_{m}\right]\tau \right\},\left(n\;+\;1/4\right)T_{m}\leq t<\left(n\;+\;3/4\right)T_{m}, \end{array}\right.$$

which could be simplified as Equation (6), considering the time delay $\tau =\frac{2r}{c}$.

(6) $$s_{b}\left(t\right)\;=\;\left\{\begin{array}{c} u_{b}\cos\left\{2\pi \left(\frac{4\Delta F}{T_{m}}\frac{r}{c}-f_{d}\right)\left(t\;-\;nT_{m}\right)\;+\;{\hat{\varphi }}_{1}^{n}\right\},\;\;\;\;\left(n-1/4\right)T_{m}\leq t<\left(n\;+\;1/4\right)T_{m}\\ u_{b}\cos\left\{2\pi \left(\frac{4\Delta F}{T_{m}}\frac{r}{c}+f_{d}\right)\left(t\;-\;nT_{m}\right)\;+\;{\hat{\varphi }}_{2}^{n}\right\},\;\;\;\left(n\;+\;1/4\right)T_{m}\leq t<\left(n\;+\;3/4\right)T_{m}, \end{array}\right.$$

where ${\hat{\varphi }}_{1}^{n}$, ${\hat{\varphi }}_{2}^{n}$ are the initial phases for the regular intervals $T_{1}$ and $T_{3}$, respectively, as expressed in Equation (7), where the Doppler frequency is $f_{d}=\frac{2V_{r}f_{c}}{c}$:

(7) $$\begin{array}{c} {\hat{\varphi }}_{1}^{n}=4\pi \left(f_{c}+f_{d}\right)\frac{r}{c}-\frac{8\pi \Delta F}{T_{m}}\frac{r^{2}}{c^{2}}-2\pi f_{d}nT_{m}\\ {\hat{\varphi }}_{2}^{n}=-4\pi \left(f_{c}+\Delta F+f_{d}\right)\frac{r}{c}+\frac{8\pi \Delta F}{T_{m}}\frac{r^{2}}{c^{2}}+2\pi f_{d}nT_{m}. \end{array}$$

### 2.3. Proposed Time-Domain Ranging Algorithm

The differentiation of the time-domain beat frequency signal $s_{b}\left(t\right)$ can be presented as

(8) $$s_{b}^{\prime }\left(t\right)\;=\;\left\{\begin{array}{c} -u_{b}2\pi \left(\frac{4\Delta F}{T_{m}}\frac{r}{c}-f_{d}\right)\sin\left\{2\pi \left(\frac{4\Delta F}{T_{m}}\frac{r}{c}-f_{d}\right)\left(t-nT_{m}\right)+{\hat{\varphi }}_{1}^{n}\right\},\left(n-1/4\right)T_{m}\leq t<\left(n+1/4\right)T_{m}\\ -u_{b}2\pi \left(\frac{4\Delta F}{T_{m}}\frac{r}{c}+f_{d}\right)\sin\left\{2\pi \left(\frac{4\Delta F}{T_{m}}\frac{r}{c}+f_{d}\right)\left(t-nT_{m}\right)+{\hat{\varphi }}_{2}^{n}\right\},\left(n+1/4\right)T_{m}\leq t<\left(n+3/4\right)T_{m}. \end{array}\right.$$

Considering an interval of the beat frequency from the regular interval, we define its duration as $T_{s}\leq T_{1}\left(T_{3}\right)$. The initial time of this interval is denoted as $T_{x}$, in which case the time frame can be expressed as $T_{x}\leq t\leq T_{x}+T_{s}$. In $T_{1}$ and $T_{3}$, the ratios of the integrals of the absolute values of $s_{b}^{\prime }\left(t\right)$ and $s_{b}\left(t\right)$ can be expressed as follows

(9) $$\frac{\int_{T_{x}}^{T_{x}+T_{s}}\left|s_{b}^{\prime }\left(t\right)\right|dt}{\int_{T_{x}}^{T_{x}+T_{s}}\left|s_{b}\left(t\right)\right|dt}=2\pi \left(\frac{4\Delta F}{T_{m}}\frac{r}{c}-f_{d}\right)\frac{\int_{T_{x}}^{T_{x}+T_{s}}\left|\sin(2\pi \left(\frac{4\Delta F}{T_{m}}\frac{r}{c}-f_{d}\right)t+{\hat{\varphi }}_{1})\right|dt}{\int_{T_{x}}^{T_{x}+T_{s}}\left|\cos(2\pi \left(\frac{4\Delta F}{T_{m}}\frac{r}{c}-f_{d}\right)t+{\hat{\varphi }}_{1})\right|dt},$$

(10) $$\frac{\int_{T_{x}+T_{m}/2}^{T_{x}+T_{s}+T_{m}/2}\left|s_{b}^{\prime }\left(t\right)\right|dt}{\int_{T_{x}+T_{m}/2}^{T_{x}+T_{s}+T_{m}/2}\left|s_{b}\left(t\right)\right|dt}=2\pi \left(\frac{4\Delta F}{T_{m}}\frac{r}{c}+f_{d}\right)\frac{\int_{T_{x}+T_{m}/2}^{T_{x}+T_{s}+T_{m}/2}\left|\sin(2\pi \left(\frac{4\Delta F}{T_{m}}\frac{r}{c}+f_{d}\right)t+{\hat{\varphi }}_{2})\right|dt}{\int_{T_{x}+T_{m}/2}^{T_{x}+T_{s}+T_{m}/2}\left|\cos(2\pi \left(\frac{4\Delta F}{T_{m}}\frac{r}{c}+f_{d}\right)t+{\hat{\varphi }}_{2})\right|dt}.$$

Equations (9) and (10) represent the ratios of those two functions in only one regular interval. If we extend the integration into *N* regular intervals, Equations (9) and (10) can be rewritten as

(11) $$\frac{\sum_{n=1}^{N}\int_{T_{x}}^{T_{x}+T_{s}}\left|s_{b}^{\prime }\left(n,t\right)\right|dt}{\sum_{n=1}^{N}\int_{T_{x}}^{T_{x}+T_{s}}\left|s_{b}\left(n,t\right)\right|dt}=2\pi \left(\frac{4\Delta F}{T_{m}}\frac{r}{c}-f_{d}\right)\Lambda_{1},$$

and

(12) $$\frac{\sum_{n=1}^{N}\int_{T_{x}+T_{m}/2}^{T_{x}+T_{s}+T_{m}/2}\left|s_{b}^{\prime }\left(n,t\right)\right|dt}{\sum_{n=1}^{N}\int_{T_{x}+T_{m}/2}^{T_{x}+T_{s}+T_{m}/2}\left|s_{b}\left(n,t\right)\right|dt}=2\pi \left(\frac{4\Delta F}{T_{m}}\frac{r}{c}+f_{d}\right)\Lambda_{2},$$

where $s_{b}\left(n,t\right)$ is the *n*th regular area of the beat frequency signal with

(13) $$\Lambda_{1}=\frac{\sum_{n=1}^{N}\int_{T_{x}}^{T_{x}+T_{s}}\left|\sin(2\pi \left(\frac{4\Delta F}{T_{m}}\frac{r}{c}-f_{d}\right)t+{\hat{\varphi }}_{1}^{n})\right|dt}{\sum_{n=1}^{N}\int_{T_{x}}^{T_{x}+T_{s}}\left|\cos(2\pi \left(\frac{4\Delta F}{T_{m}}\frac{r}{c}-f_{d}\right)t+{\hat{\varphi }}_{1}^{n})\right|dt},$$

and

(14) $$\Lambda_{2}=\frac{\sum_{n=1}^{N}\int_{T_{x}+T_{m}/2}^{T_{x}+T_{s}+T_{m}/2}\left|\sin(2\pi \left(\frac{4\Delta F}{T_{m}}\frac{r}{c}+f_{d}\right)t+{\hat{\varphi }}_{2}^{n})\right|dt}{\sum_{n=1}^{N}\int_{T_{x}+T_{m}/2}^{T_{x}+T_{s}+T_{m}/2}\left|\cos(2\pi \left(\frac{4\Delta F}{T_{m}}\frac{r}{c}+f_{d}\right)t+{\hat{\varphi }}_{2}^{n})\right|dt},$$

where ${\hat{\varphi }}^{n}$ denotes the initial phase of the *n*th regular area. Combining the *N* regular interval ratios Equations (11) and (12), we have

(15) $$\begin{array}{cc} \frac{\sum_{n=1}^{N}\int_{T_{x}}^{T_{x}+T_{s}}\left|s_{b}^{\prime }\left(n,t\right)\right|dt}{\sum_{n=1}^{N}\int_{T_{x}}^{T_{x}+T_{s}}\left|s_{b}\left(n,t\right)\right|dt}+\frac{\sum_{n=1}^{N}\int_{T_{x}+T_{m}/2}^{T_{x}+T_{s}+T_{m}/2}\left|s_{b}^{\prime }\left(n,t\right)\right|dt}{\sum_{n=1}^{N}\int_{T_{x}+T_{m}/2}^{T_{x}+T_{s}+T_{m}/2}\left|s_{b}\left(n,t\right)\right|dt} & =8\pi \frac{\Delta F}{T_{m}c}r\left(\Lambda_{2}+\Lambda_{1}\right)+2\pi f_{d}\left(\Lambda_{2}-\Lambda_{1}\right)\\ & =2\pi \frac{4\Delta F}{T_{m}c}r\left(\Lambda_{2}+\Lambda_{1}\right)+2\pi f_{d}\left(\Lambda_{2}-\Lambda_{1}\right). \end{array}$$

When the value of $T_{s}$ and *N* are large enough, the value of Λ can get close to 1 [19]. Based on Equation (13) and Λ≈1, we propose the range estimation $\hat{r}$ as

(16) $$\hat{r}=\frac{1}{4\pi C}\left(\frac{\sum_{n=1}^{N}\int_{T_{x}}^{T_{x}+T_{s}}\left|s_{b}^{\prime }\left(n,t\right)\right|dt}{\sum_{n=1}^{N}\int_{T_{x}}^{T_{x}+T_{s}}\left|s_{b}\left(n,t\right)\right|dt}+\frac{\sum_{n=1}^{N}\int_{T_{x}+T_{m}/2}^{T_{x}+T_{s}+T_{m}/2}\left|s_{b}^{\prime }\left(n,t\right)\right|dt}{\sum_{n=1}^{N}\int_{T_{x}+T_{m}/2}^{T_{x}+T_{s}+T_{m}/2}\left|s_{b}\left(n,t\right)\right|dt}\right),\;\;\;\;\;\;C=\frac{4\Delta F}{T_{m}c},$$

where C is the product of a constant and signal frequency slope $\Delta F/T_{m}$, which indicates the linearity of beat frequency signal. The ranging resolution of this proposed time-domain method will be also impacted by the frequency bandwidth ΔF. We will analyze the ranging error in the following section.

## 3. Performance of Proposed Ranging Algorithm

### 3.1. Analysis of Ranging Error

The inherent error of the proposed estimation algorithm Equation (16) can be calculated as

(17) $$\begin{array}{cc} e=r\;-\;\hat{r} & =r-\frac{1}{4\pi C}\left[2\pi Cr\left(\Lambda_{1}+\Lambda_{2}\right)+2\pi f_{d}\left(\Lambda_{2}-\Lambda_{1}\right)\right]\\ & =r\left(1-\frac{\Lambda_{1}+\Lambda_{2}}{2}\right)+\frac{f_{d}}{2C}\left(\Lambda_{2}-\Lambda_{1}\right). \end{array}$$

Under the assumption that $g\left(\varphi \right)=\int_{0}^{\varphi }\left|\cos\varphi \right|d\varphi$, we can derive the numerator and denominator from Equation (13) as

(18) $$\int_{T_{x}}^{T_{x}+T_{s}}\left|\sin\left[2\pi \left(Cr-f_{d}\right)t\right]+{\hat{\varphi }}_{1}^{n}\right|dt=\frac{1}{2\pi (Cr-f_{d})}\left[g\left(\varphi_{T_{x}+T_{s}}^{n}-\frac{\pi }{2}\right)-g\left(\varphi_{T_{x}}^{n}-\frac{\pi }{2}\right)\right],$$

and

(19) $$\int_{T_{x}}^{T_{x}+T_{s}}\left|\cos\left[2\pi \left(Cr-f_{d}\right)t\right]+{\hat{\varphi }}_{1}^{n}\right|dt=\frac{1}{2\pi (Cr-f_{d})}\left[g\left(\varphi_{T_{x}+T_{s}}^{n}\right)-g\left(\varphi_{T_{x}}^{n}\right)\right].$$

Therefore, we arrive at

(20) $$\Lambda_{1}=\frac{\sum_{n=1}^{N}\left[g\left(\varphi_{T_{x}+T_{s}}^{n}-\frac{\pi }{2}\right)-g\left(\varphi_{T_{x}}^{n}-\frac{\pi }{2}\right)\right]}{\sum_{n=1}^{N}\left[g\left(\varphi_{T_{x}+T_{s}}^{n}\right)-g\left(\varphi_{T_{x}}^{n}\right)\right]},$$

where

(21) $$\varphi_{T_{x}+T_{s}}^{n}=2\pi \left(Cr-f_{d}\right)\left(T_{x}+T_{s}\right)+{\hat{\varphi }}_{1}^{n},$$

(22) $$\varphi_{T_{x}}^{n}=2\pi \left(Cr-f_{d}\right)\left(T_{x}\right)+{\hat{\varphi }}_{1}^{n}.$$

The approximate solution of g(φ) [19] can be expressed as

(23) $$g\left(\varphi \right)=\frac{2}{\pi }\varphi +0.2105\sin\left(2\varphi \right),$$

from which we can expand it as,

(24) $$\begin{array}{cc} g\left(\varphi_{T_{x}+T_{s}}^{n}\right)-g\left(\varphi_{T_{x}}^{n}\right) & =4\left(Cr-f_{d}\right)T_{s}+0.421\sin\left[2\pi \left(Cr-f_{d}\right)T_{s}\right]\cos\left[2\pi \left(Cr-f_{d}\right)\left(T_{s}+2T_{x}\right)+2{\hat{\varphi }}_{1}^{n}\right], \end{array}$$

and

(25) $$\begin{array}{cc} g\left(\varphi_{T_{x}+T_{s}}^{n}-\frac{\pi }{2}\right)-g\left(\varphi_{T_{x}}^{n}-\frac{\pi }{2}\right) & =4\left(Cr-f_{d}\right)T_{s}-0.421\sin\left[2\pi \left(Cr-f_{d}\right)T_{s}\right]\cos\left[2\pi \left(Cr-f_{d}\right)\left(T_{s}+2T_{x}\right)+2{\hat{\varphi }}_{1}^{n}\right]. \end{array}$$

Therefore, we can update $\Lambda_{1}$ from Equation (20) as

(26) $$\Lambda_{1}=\frac{4\left(Cr-f_{d}\right)T_{s}N-0.421\sin\left[2\pi \left(Cr-f_{d}\right)T_{s}\right]\sum_{n=1}^{N}\cos\left[2\pi \left(Cr-f_{d}\right)\left(T_{s}+2T_{x}\right)+2{\hat{\varphi }}_{1}^{n}\right]}{4\left(Cr-f_{d}\right)T_{s}N+0.421\sin\left[2\pi \left(Cr-f_{d}\right)T_{s}\right]\sum_{n=1}^{N}\cos\left[2\pi \left(Cr-f_{d}\right)\left(T_{s}+2T_{x}\right)+2{\hat{\varphi }}_{1}^{n}\right]}.$$

Similarly, we can obtain $\Lambda_{2}$ as

(27) $$\Lambda_{2}=\frac{4\left(Cr+f_{d}\right)T_{s}N-0.421\sin\left[2\pi \left(Cr+f_{d}\right)T_{s}\right]\sum_{n=1}^{N}\cos\left[2\pi \left(Cr+f_{d}\right)\left(T_{s}+2T_{x}+T_{m}\right)+2{\hat{\varphi }}_{1}^{n}\right]}{4\left(Cr+f_{d}\right)T_{s}N+0.421\sin\left[2\pi \left(Cr+f_{d}\right)T_{s}\right]\sum_{n=1}^{N}\cos\left[2\pi \left(Cr+f_{d}\right)\left(T_{s}+2T_{x}+T_{m}\right)+2{\hat{\varphi }}_{1}^{n}\right]}.$$

Finally, we have the error *e* of the proposed ranging algorithm as

(28) $$\begin{array}{cc} e= & \frac{0.421}{4CT_{s}N}\sin\left[2\pi \left(Cr-f_{d}\right)T_{s}\right]\sum_{n=1}^{N}\cos\left[2\pi \left(Cr-f_{d}\right)\left(T_{s}+2T_{x}\right)+{\hat{\varphi }}_{1}^{n}\right]\\ & +\sin\left[2\pi \left(Cr+f_{d}\right)T_{s}\right]\sum_{n=1}^{N}\cos\left[2\pi \left(Cr+f_{d}\right)\left(T_{s}+2T_{x}+T_{m}\right)+{\hat{\varphi }}_{1}^{n}\right]. \end{array}$$

### 3.2. Ranging Error Performance

**Figure 4:**
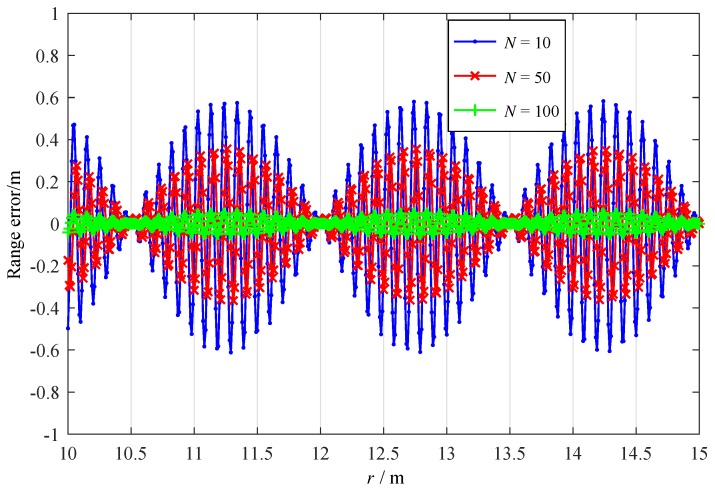
Range error versus *r* when *N* = 10, 50, 100; and $v_{r}=$ 10 m/s.

According to Equation (28), the range error of proposed time-domain ranging algorithm is related to the number of intervals *N* in the intercepted beat frequency signal, the real range *r*, and the relative speed $v_{r}$. The range error variations for increasing range *r* are investigated in Figure 4 with N= 10, 50, 100, and $v_{r}$ = 10 m/s. Simulation results indicate that as *N* increases, the range error decreases, e.g., the range error gets close to zero for N= 100. We notice that, range error *e* is a periodic function of *r* with a period of $1/CT_{s}$. In our simulations, the range error changes periodically with a period r= 1.5 m.

**Figure 5:**
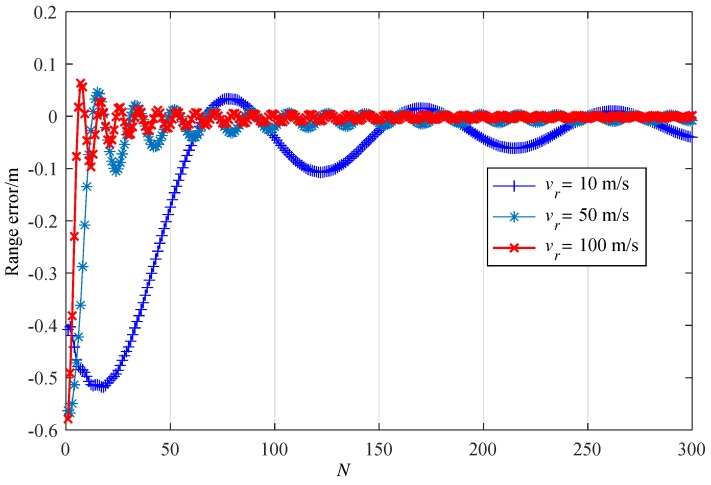
Range error versus *N* when $v_{r}$ = 10, 50, 100 m/s; and r= 10 m.

The range error performance for increasing *N* is examined in Figure 5 with three different relative velocities $v_{r}=$ 10 m/s, 50 m/s, 100 m/s; and range of r= 10 m. Simulation results indicate that the range error of the proposed algorithm could be significantly decreased with increasing *N* and it will converge around zero for all velocities. Furthermore, for $v_{r}=$ 10 m/s, 50 m/s, 100 m/s, the range errors decrease and cross zeros with integral N= 67, 13, 6, respectively. The larger the value of $v_{r}$, the faster the phase of the sum can get through, which correspondingly makes the value of the sum reduce at a higher rate and finally accelerates the reduction of the range error.

**Figure 6:**
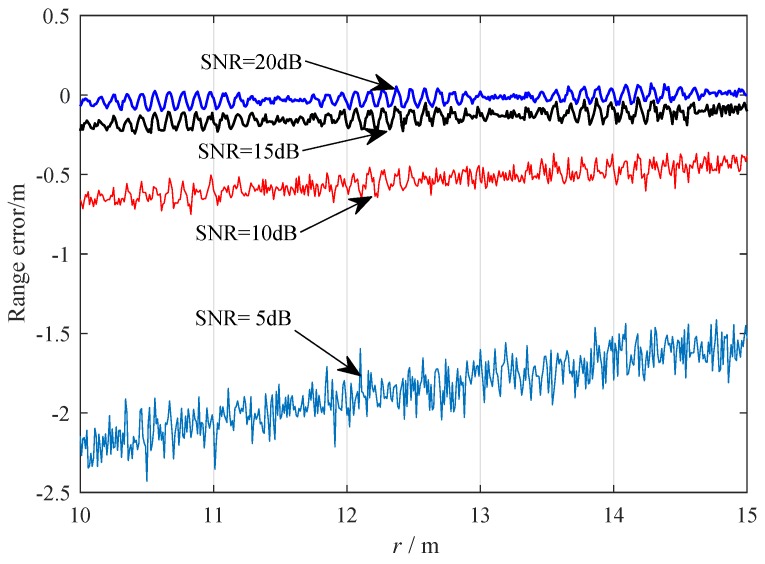
Range error versus *r* when signal-to-noise ratios (SNR) = 5 dB, 10 dB, 15 dB, 20 dB.

To analyze the influence of the signal-to-noise ratio (SNR) on the range error, we conducted simulations for the time-domain ranging method based on derivative ratio with ΔF= 50 MHz, $T_{m}\;=$ 10 μs, $f_{c}=$ 8.2 GHz, $v_{r}=$ 10 m/s, and N= 20. As Figure 6 shows, increasing SNR from 5 dB to 20 dB can effectively improve the range accuracy of the time-domain ranging algorithm. At *r* = 15 m, for SNR = 5 dB, 10 dB, 15 dB, 20 dB; and the range errors are −1.48 m, −0.40 m, −0.11 m, −0.04 m, respectively. There is a periodic function of *r*, which is consistent with the result showed in Figure 4. For low SNR, the noise would obscure this periodicity. From *r* = 10 m to *r* = 15 m, three complete periodicities can be discriminated in our simulations.

### 3.3. Computational Load Analysis

From Equations (11) and (14), we can find that the proposed time-domain ranging algorithm requires one derivative and two integrations, which are equivalent to $NN_{T_{s}}$ subtractions, for *N* regular interval with $N_{T_{s}}$ samples in each regular interval. Therefore, the total complexity imposed by the proposed algorithm is $3NN_{T_{s}}$ subtractions. Under the assumption with $M=NN_{T_{s}}$ samples, the FFT algorithm needs $6M\log_{2}M$ additions and $6M\log_{2}M$ multiplications in total. The computational load is mainly determined by the number of multiplication operations in ranging algorithms. The critical difference between the proposed time-domain algorithm and the conventional FFT algorithm lies in that the time-domain algorithm eliminates the requirements of complex multiplication operations. The computational loads of both FFT estimation and the proposed algorithm are compared in Table 1.

## 4. Implementation and Experiment Results

### 4.1. FMCW Sensor Prototype

**Figure 7:**
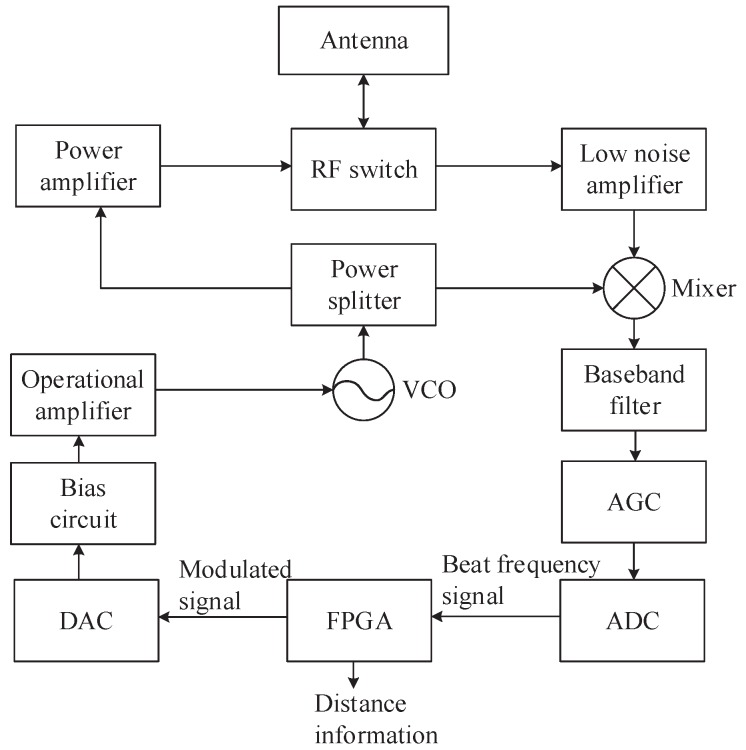
Block diagram of the frequency-modulated continuous wave (FMCW) ranging system.

We construct an FMCW ranging system following the architecture presented in Figure 7. The ranging system is designed as follows.

- We generate the digital modulation signal in the field-programmable gate array (FPGA) and convert it to analog signal by the digital analog converter (DAC). Then, we adjust its bias voltage and signal amplitude by the bias circuit and the operational amplifier, respectively.
- The voltage controlled oscillator (VCO) is used to generate the modulated high-frequency radar signal, which is subsequently divided into two identical signals by a power splitter. One branch of radar signal is amplified by a power amplifier and sent to the antenna for transmission through radio frequency (RF) switch, which provides high isolation between the transmission and detection of the radio signal to avoid mutual signal leakage. In the mixer, the signal received from the microstrip antenna is amplified through low noise amplifier (LNA) and then mixed by the other branch of radar signal to obtain the beat frequency signal.
- The beat frequency signal will finally be sent to FPGA for signal processing after baseband filtering, amplification, and adjustment. The range will be calculated by the proposed ranging algorithm (16).

**Figure 8:**
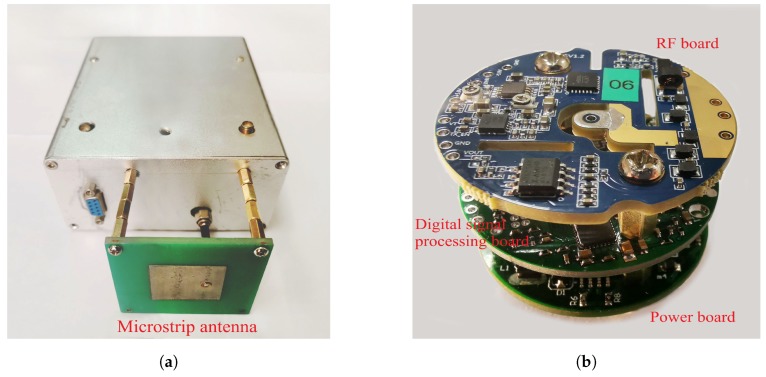
FMCW sensor prototype. (**a**) Sensor structure and microstrip antenna. (**b**) Radio frequency (RF) front-end and signal processing module.

The components of FMCW sensor prototype are given in Figure 8, where Figure 8a shows the sensor structure and microstrip antenna, which is vertically polarized with omnidirectional pattern and a gain of 6 dB in half beamwidth. The RF front-end and signal processing modules mounted inside the sensor structure are fabricated as Figure 8b. The specifications of FMCW sensor are summarized in Table 2.

### 4.2. Experiment and Results

**Figure 9:**
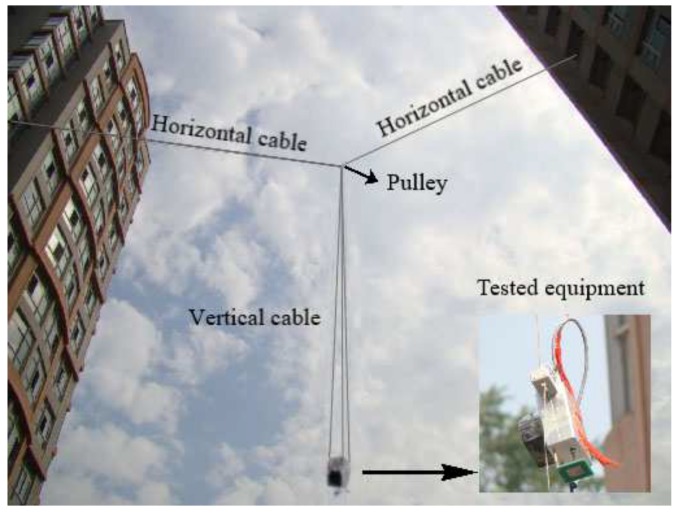
Experimental setup of the ranging system.

In order to study the performance of the designed time-domain ranging system, we conducted an experiment at the campus of the Beijing Institute of Technology, as shown in Figure 9. The experimental setup consists of one horizontal cable, three vertical cables, and the tested equipment.

One of the vertical cables is placed with a Hall sensor every 0.2 m. The horizontal cable is fixed between the two tall buildings at campus. The pulley is fixed in the middle of the horizontal cable with a height of 18 m. The experimented equipment falls down with approximate speed of 10 m/s from altitudes of 18 m to 4 m. The tested equipment plays two roles: (1) Outputting the enable signal through the Hall sensors on the cable, which make the tested equipment record its own range to the ground once per 0.2 m; (2) receiving the signal and calculating the range to the ground using the proposed time-domain ranging algorithm. Finally, we compared these two range results and analyzed the range error therein.

**Figure 10:**
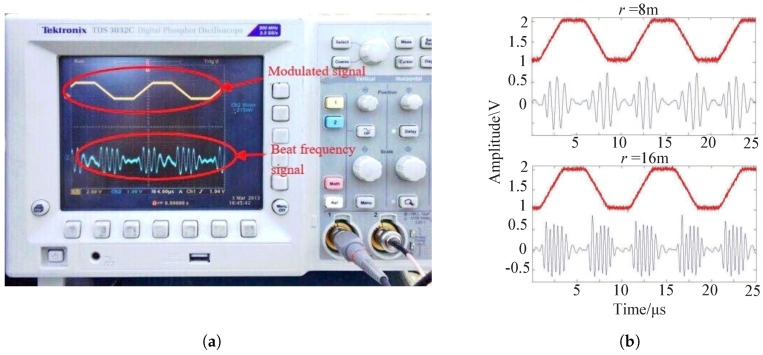


In the experiment, we employed a trapezoidal waveform as the modulation signal instead of a triangular waveform to eliminate the phase aberration in the beat signal while retaining the regular interval of the beat signal. We set modulation frequency $F_{m}$ = 100 kHz, rise and fall times of the trapezoidal waveform $T_{r}=T_{f}=5\;\mu$s, frequency bandwidth ΔF = 50 MHz, central frequency $f_{c}=$ 8.2 GHz, and sample rate $F_{s}=$ 10 MHz. We used the signal with 10 modulation periods (N= 20 regular intervals) to calculate the range and carried out the experiments 20 times. The range is from 18 m to 8 m, with a 0.2 m gap between adjacent placements. As shown in Figure 10a, the modulated signal and beat signal were measured by the oscilloscope. From the waveform of the beat signals in Figure 10b for range r= 8 m and 16 m, we can find that the beat frequency increases evidently for increasing range.

**Figure 11:**
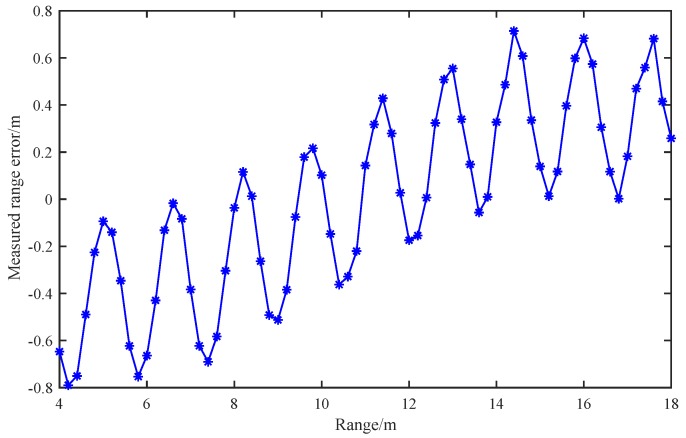
Measured range error under different ranges.

The measured range errors from our experiments are provided in Figure 11, which illustrate the relationship between the measured range errors and the real ranges. The range errors are the difference between the measured range values and the range at a speed of 10 m/s. For increasing speed, the range error drifts are observed due to the speed deviation during the experimented equipment falling down. We can find that for r= 4 m to 18 m, all the range errors fall within 0.8 m, whereas the range error exhibits periodically feature with the increasing range *r*, which is consistent with the simulation results in Figure 4. Besides, the theoretical range resolution of the conventional frequency-domain methods $\Delta R=\frac{c}{4\Delta F}$ is 1.5 m under the same experimental conditions.

## 5. Conclusions

In this paper, we proposed a time-domain ranging algorithm based on the ratio of the deviation of the beat frequency signal for single target short range sensors to improve ranging performance and reduce computation complexity. We investigated the ranging errors under different SNRs, moving speeds, integral numbers, and compare the computation complexity. A measurement system was then constructed with an FMCW sensor prototype to validate our analysis results. The time-domain ranging algorithm avoids multiplications in implementation, which allows great advantages over the conventional zero-padding FFT scheme. Meanwhile, it can achieve a range resolution of 0.8 m in our experiment. The proposed ranging method could find wide application in various scenarios of single target short range systems, whereas the multiple targets scenario is beyond the scope of this method.

## Figures and Tables

**Table 1:** Computation complexity for fast Fourier transform (FFT) and proposed time-domain algorithms with *M* samples.

Ranging Schemes	Additions/Subtractions	Multiplications	Divisions
FFT estimation	$6M\log_{2}M$	$2M\log_{2}M$	0
Proposed algorithm	3M	0	1

**Table 2:** Specifications of FMCW sensor.

Item	Value	Item	Value
Carrier frequency	8.2 GHz	Frequency bandwidth	50 MHz
Modulation waveform	trapezoidal	Tx-Rx Switching Frequency	4 MHz
Modulation frequency	100 kHz	Sample rate	10 MHz
Antenna beamwidth	100${}^{\circ }$	Antenna gain	6 dBi
